# Phylogenetic and crystallographic analysis of *Nostoc* phycocyanin having blue-shifted spectral properties

**DOI:** 10.1038/s41598-019-46288-4

**Published:** 2019-07-08

**Authors:** Ravi R. Sonani, Rajesh Prasad Rastogi, Stuti Nareshkumar Patel, Mukesh Ghanshyam Chaubey, Niraj Kumar Singh, Gagan D. Gupta, Vinay Kumar, Datta Madamwar

**Affiliations:** 1 Radiation Biology & Health Sciences Division, Bhabha Atomic Research Centre, Trombay, Mumbai, 400 085 India; 2 Ministry of Environment, Forest & Climate Change, Indira Paryavaran Bhawan, New Delhi, 110003 India; 3 Post-Graduate Department of Biosciences, Satellite Campus, Sardar Patel University, Bakrol, Anand, 388 315 Gujarat, India; 4 Shri A. N. Patel P. G. Institute of Science and Research, Sardar Patel University, Anand, Gujarat, 388001 India

**Keywords:** Protein analysis, X-ray crystallography, Molecular evolution

## Abstract

The distinct sequence feature and spectral blue-shift (~10 nm) of phycocyanin, isolated from *Nostoc* sp. R76DM (N-PC), were investigated by phylogenetic and crystallographic analyses. Twelve conserved substitutions in N-PC sequence were found distributed unequally among α- and β-subunit (3 in α- and 9 in β-subunit). The phylogenetic analysis suggested that molecular evolution of α- and β-subunit of *Nostoc*-phycocyanin is faster than evolution of *Nostoc*-species. The divergence events seem to have occurred more frequently in β-subunit, compared to α-subunit (relative divergence, 7.38 for α-subunit and 9.66 for β-subunit). Crystal structure of N-PC was solved at 2.35 Å resolution to reasonable R-factors (R_work_/R_Free_ = 0.199/0.248). Substitutions congregate near interface of two αβ-monomer in N-PC trimer and are of compensatory nature. Six of the substitutions in β-subunit may be involved in maintaining topology of β-subunit, one in inter-monomer interaction and one in interaction with linker-protein. The β153Cys-attached chromophore adopts high-energy conformational state resulting due to reduced coplanarity of B- and C-pyrrole rings. Distortion in chromophore conformation can result in blue-shift in N-PC spectral properties. N-PC showed significant *in*-*vitro* and *in*-*vivo* antioxidant activity comparable with other phycocyanin. Since *Nostoc*-species constitute a distinct phylogenetic clade, the present structure would provide a better template to build a model for phycocyanins of these species.

## Introduction

Phycobiliproteins (PBPs) are accessory light harvesting, water-soluble and fluorescence proteins present in cyanobacteria, red algae and cryptophyte^1^. They form a light harvesting complex called phycobilisome in association with colorless linker proteins^2^. PBPs harvest light in the region 500–655 nm of solar spectrum, which could not be absorbed by chlorophyll^3,4^. In order to absorb light in this region, PBPs harbor chromophores having an open chain tetrapyrrole structure called phycobilins^2^. Based on the chemical and spectral properties, phycobilins are divided into four groups, phycocyanobilin (PCB), phycoerythrobilin (PEB), phycoviolobilin (PVB) and phycourobilin (PUB)^5^. The PBP, by acquiring one or more types of phycobilin commences distinct light absorption capacity. Based on absorption properties, PBPs are categorized into four main groups, phycoerythrin (PE, λ_max_ = 540–570 nm), phycocyanin (PC, λ_max_ = 615–620 nm), phycoerythrocyanin (PEC, λ_max_ = 575 nm) and allophycocyanin (APC, λ_max_ = 650–655 nm)^2,6^. Specific spatial arrangement and interactions of phycobilin with surrounding protein introduce further spectral diversity among same group of PBPs isolated from different organisms^7,8^. For instance, the PCs isolated from *Phormidium rubidum* A09DM^9^ and *Gloeobacter violaceus*^10^ have different absorbance maxima, 616 and 620 nm, respectively (Suppl. Material I). This flexibility allows cyanobacteria to survive across diverse habitats with fluctuating light environment. In order to understand the fashion of spectral variation among PBPs of different origin, knowledge of the phycobilin-apoprotein interactions is crucial. Besides their role in cyanobacteria, PBPs have been widely documented to possess the antioxidant, hepatoprotective, neuroprotective, anti-inflammatory and anti-aging activity^11,12^.

The *Nostoc* sp. R76DM is a filamentous cyanobacterium, which was isolated from the central part of Gujarat, India^13^. The PBPs isolated from *Nostoc* sp. R76DM display unique composition and spectral properties, as compared to reported PBPs^14^. Unlike other PCs (from *Arthrospira platensis*, *Spirulina* sp., *Leptolyngbya* sp., *Phormidium rubidum* A09DM, *Galdieria sulphuraria* and *others*), the PC of *Nostoc* sp. R76DM (N-PC) acquires distinct sequence feature and unique blue-shifted spectral properties (Suppl. Material I) that makes it an important variant to study. The present report describes physico-chemical, phylogenetic and structural analysis of N-PC. The study provides structural implication of distinct sequence features, structural attributes responsible for N-PC’s unique spectral properties and possible correlation between them. Furthermore, the effect of distinct sequence features on an antioxidant activity of N-PC is accessed *in*-*vitro* and *in*-*vivo*.

## Results

### Physico-chemical analysis

**Figure 1:**
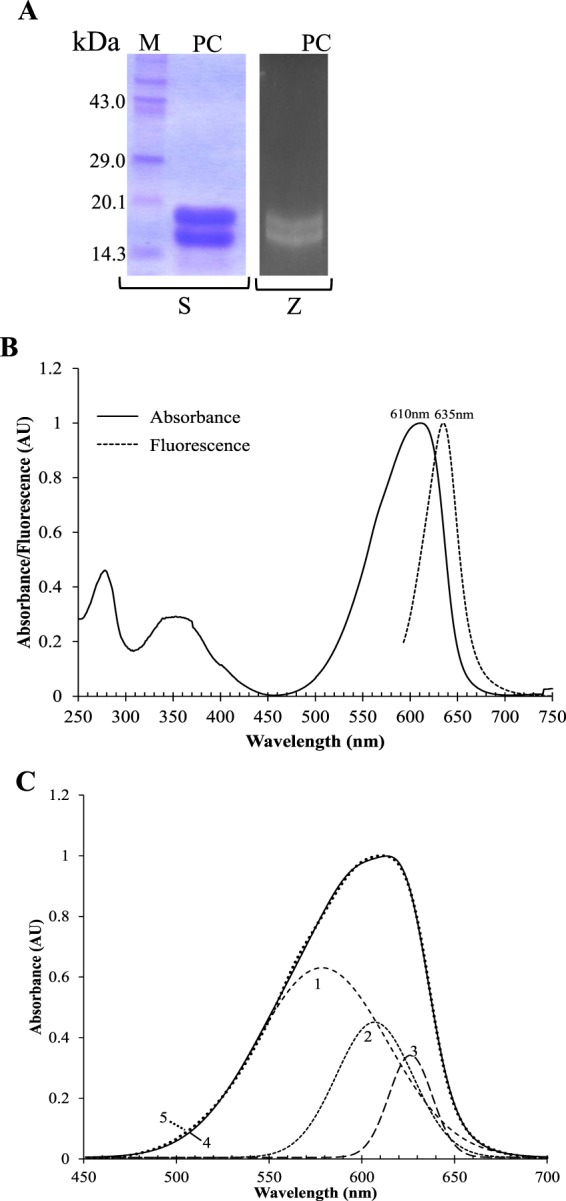
Physico-chemical analysis of *Nostoc* sp. R76DM phycocyanin (N-PC). (**A**) SDS-PAGE profiles of purified N-PC. M, Molecular weight marker; PC, Purified N-PC; S, Silver-stained; Z, Zinc acetate stained. (**B**) Steady state UV-visible (Intact line) and fluorescence emission (Dashed line) spectra of purified N-PC. Fluorescence emission spectra were recorded upon exciting the N-PC at 580 nm. (**C**) Deconvoluated absorption spectrum of N-PC having three (denoted by 1, 2, and 3) Gaussian components. The Gaussian-fitted (sum of deconvoluated components) and experimental absorption spectra are denoted as 4 and 5, respectively.

PBPs consist of two peptide chains designated as α- and β-subunit^2,6^. Silver-stained SDS-PAGE of purified N-PC showed two bands near 18 kDa, corresponding to α- and β-subunit (Fig. 1A). Presence of phycocyanobilin (PCB) with N-PC subunits was confirmed by chromophore specific zinc acetate staining (Fig. 1A). UV-visible absorbance profile of N-PC showed an absorption maximum at 611 nm (Fig. 1B), which is blue shifted as compared to reported PCs (Suppl. Material I). The functional integrity (fluorescence emission capacity) of N-PC was accessed by recording its fluorescence emission spectrum with an excitation at 580 nm. The emission spectrum is also blue-shifted with peak at 635 nm as compared to reported PCs (Fig. 1B) (Suppl. Material I). Analysis of N-PC absorption spectrum derivative suggested that it is composite spectra of more than one peak. The deconvolution of N-PC absorbance spectrum gave a best-fitted model containing three Gaussian components (named 1, 2 and 3) with an absorbance maximum at 578, 607 and 626 nm, respectively (Fig. 1C). The PC of *Mastigocladus laminosus* (hereafter ML-PC) has also documented to possess three Gaussian absorption components, 598, 618 and 625 nm^15^. Comparison of N-PC and ML-PC absorption components suggested that component 1 and 2 are blue-shifted by ~20 nm and ~11 nm, respectively, in N-PC as compared to ML-PC.

### Sequence and phylogenetic analysis

The amino acid sequence of N-PC was analyzed with reference to other PC by multiple sequence alignment (Suppl. Material II). Twelve residues are noticed to be conservatively substituted in N-PC (Table 1). Despite the coding genes of both PC subunits (*cpcA* and *cpcB*) are placed closely on genome, the frequency of substitutions in α- and β-subunit is not uniform, as only three substitutions belong to α-subunit whereas nine belong to β-subunit (Table 1). Substitutions found in *Nostoc* sp. R76DM are also observed in other *Nostoc* species. For instance, *Nostoc linckia*, *Nostoc* sp. *Lobaria pulmonaria* and *Nostoc* sp. CENA543 show substitution at 9, 8 and 6 positions out of 9 found in *Nostoc* sp. R76DM β-subunit (Suppl. Material II). It can thus be thought that an additional evolutionary pressure might be responsible for biased substitutions in PC β-subunit of *Nostoc* species.

**Table 1:** List of substitutions found in *Nostoc* sp. R76DM phycocyanin sequences.

Subunit	Position	Conserved residue	Substituted in N-PC
α	18	Phe (F)	Tyr (Y)
	88	Ile (I)	Val (V)
	98	Cys (C)	Ala (A)
β	56	Ala (A)	Val (V)
	60	Phe (F)	Trp (W)
	179	Met (M)	Ala (A)
	183	Leu (L)	Ile (I)
	186	Met (M)	Leu (L)
	187	Glu (E)	Asp (D)
	109	Cys (C)	Ala (A)
	134	Met (M)	Leu (L)
	138	Ala (A)	Ser (S)

**Figure 2:**
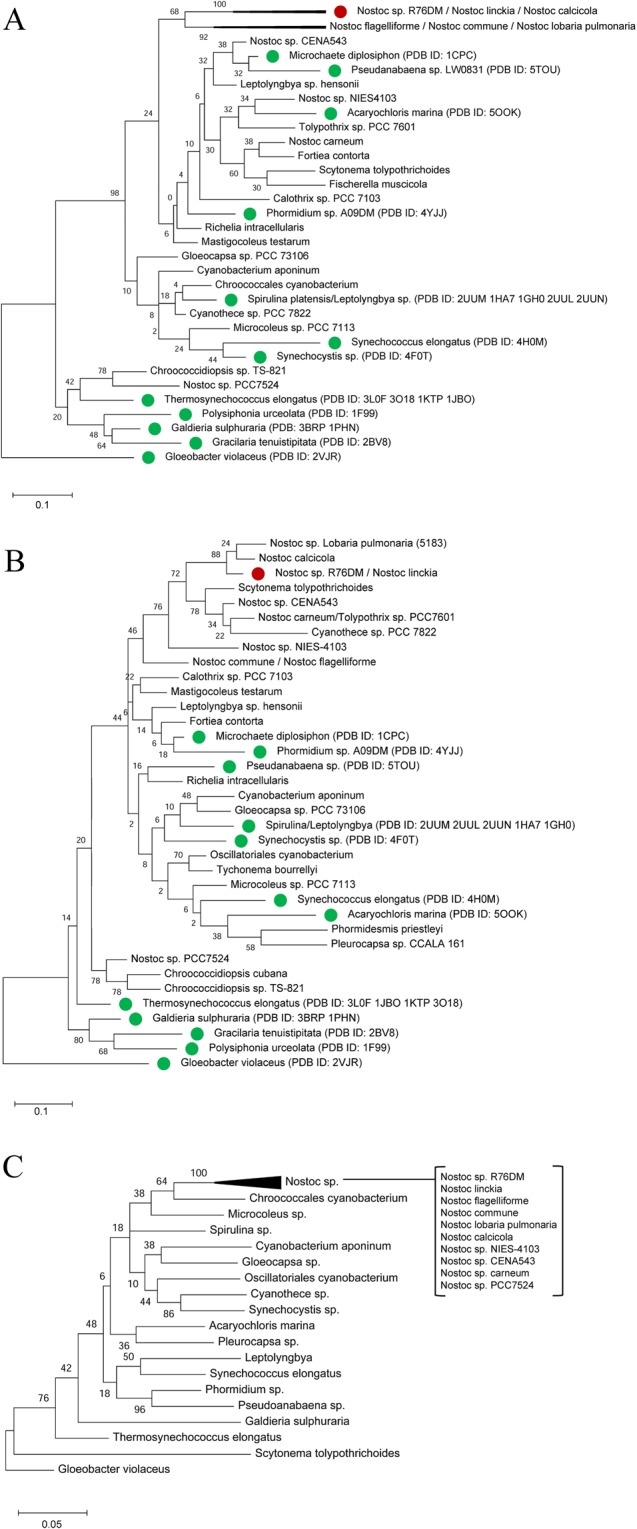
Phylogenetic tree of phycocyanin α- (**A**) and β-subunit (**B**), and 16S rRNA gene (**C**) of cyanobacterial species. For taxa, denoted with green-filled circles, phycocyanin structure are available in the PDB. The evolutionary history was inferred by using the Maximum Likelihood method and JTT matrix-based model^23^. The trees with the highest log likelihood are shown. The trees are drawn to scale with branch lengths measured in the number of substitutions per site. Evolutionary analyses were conducted in MEGA X^22^.

To check the relative evolutionary nature of N-PC α- and β-subunits, cyanobacterial phylogeny based on PC α- and β-subunit amino acid sequence, and 16S rRNA gene sequence have been constructed using sequences available in NCBI. In all phylogeny reconstructions (Fig. 2), the *Nostoc* sp. R76DM is clustered in a lately diverged clade representing *Nostoc* family. Interestingly, the analyses also suggest that *Nostoc* species are not clustered in single clade in α- and β-subunit (Fig. 2A,B) as they do in 16S rRNA gene based phylogeny (Fig. 2C). This may suggest that both PC subunits are diverging with a rate faster that an evolutionary rate of *Nostoc* species. Relative divergence times (calculated excluding common extreme outliers) of 7.38 for α-subunit and 9.66 for β-subunit suggest that the divergence events have occurred more frequently in β-subunit as compared to α-subunit of *Nostoc* PCs. Since *Nostoc* has been widely documented as rapidly diverging family in response to climate change^16^, faster divergence in these genes might have happened under the pressure of maintaining light harvesting function in extreme environmental condition. Mining of available PC structures in the Protein data Bank (PDB) suggests that no PC crystal structure is available for a node from which *Nostoc* has evolved (Fig. 2).

### Crystallographic analysis

#### Crystal packing and N-PC structure

Diffraction quality crystals of N-PC were obtained using 0.2 M sodium malonate with 20% (w/v) PEG 3350 as a reservoir solution in sitting drop plates. The N-PC crystals belong to *P*2_1_ space group with unit cell parameters, a = 67.13 Å, b = 186.15 Å, c = 85.53 Å, α, γ = 90.0° and β = 94.3° and diffracted an X-ray beam up to 2.35 Å. Data collection statistics are given in Table 2. Initial phases were estimated by the molecular replacement method using *Gracilaria chilensis* PC (α_3_β_3_)_2_ hexamer (PDB ID: 2bv8) coordinates as a search model. The asymmetric unit consists of six copies of αβ heterodimer (one (α_3_β_3_)_2_ hexamer) with 48.66% solvent content. Initial phases were accurate and clearly revealed electron density for phycocyanobilin (PCB) chromophores, which were not included in search model. Initial model was refined against 2.35 Å data up to *R*_*work*_ (*R*_*free*_) value 0.199 (0.248) with reasonable stereo-chemistry (Table 2). The atomic co-ordinate and structure factors have been deposited in the Protein Data Bank (PDB) with PDB ID 6JPR.

**Table 2:** Summary of XRD data collection and structure refinement statistics of *Nostoc* sp. R76DM phycocyanin.

**Crystallographic Data statistics for Phycocyanin**	
Unit Cell	67.14, 186.16, 85.53 (Å), 90.00 94.33 90.00 (°)
Space group	*P* 2_1_
Resolution limits (Å)	46.54–2.35 (2.39–2.35)*
Solvent content (%)	48.66
Unique reflections	84711
Redundancy	2.6 (2.6)*
Completeness (%)	97.7 (99.0)*
R_merge_	0.09 (0.406)*
Mean I/mean σ(I)	8.5 (2.5)*
**Refinement statistics**	
Resolution range (Å)	37.26–2.35
Wilson B (Å^2^)	28.38
Final R_work_/R_free_	0.199/0.248
Number of non-hydrogen atoms	16694
Ramachandaran plot (favored/allowed/disallowed)	98.13/1.77/0.0
Root-mean-square deviation from ideality	
Bond lengths (Å)	0.003
Bond angles (°)	1.096

*Values given in bracket are for the highest resolution outer shell.

**Figure 3:**
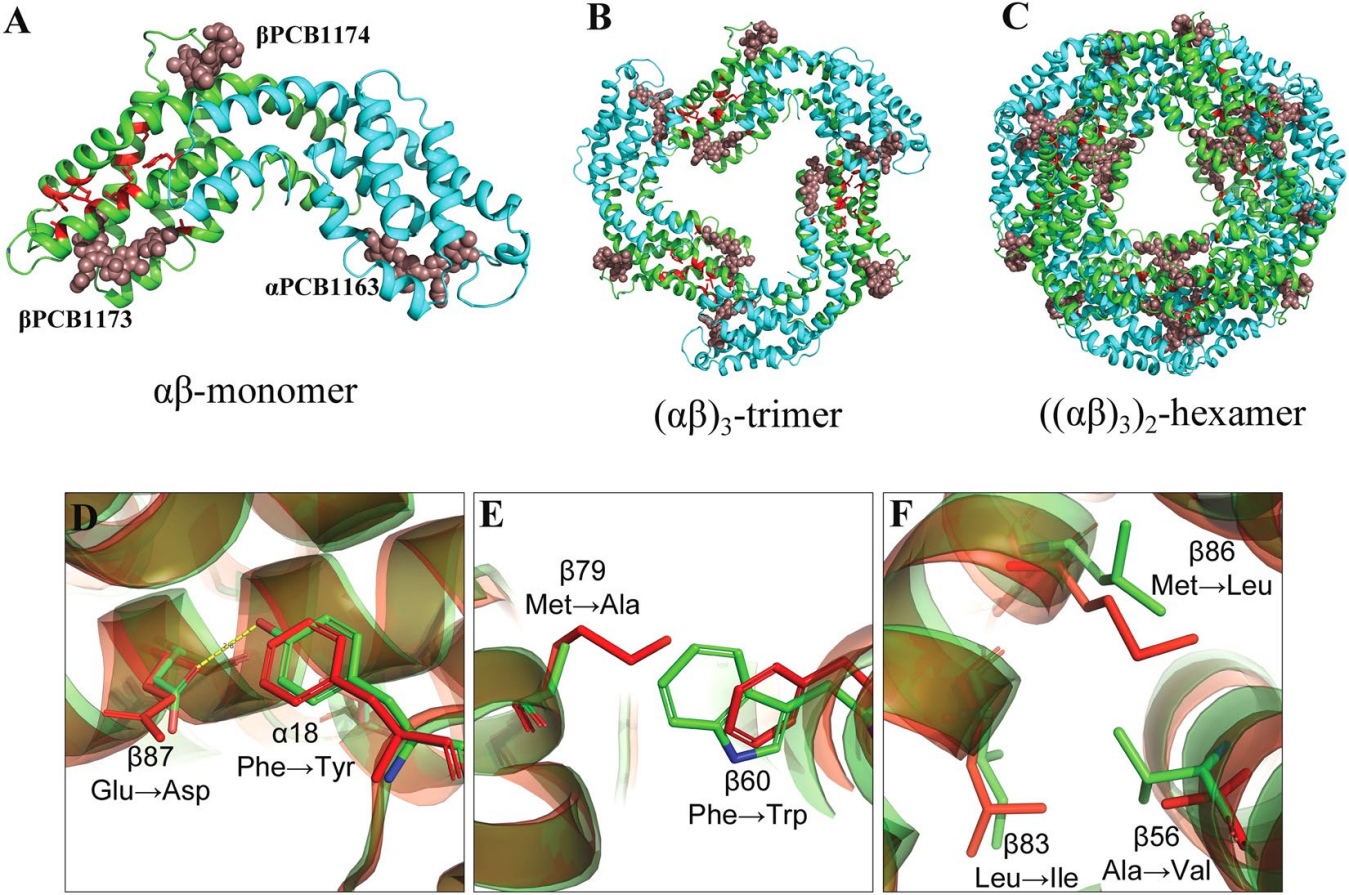
(**A**–**C**) Cartoon representation of αβ monomer (**A**), α_3_β_3_ trimer (**B**) and (α_3_β_3_)_2_ hexamer (**C**) of *Nostoc* sp. R76DM phycocyanin. The α- and β-subunits are represented by cyan and green colors, respectively. PCB chromophores are presented in brown color. Substituted residues of β-subunit are represented by red colour. (**D**–**F**) Superimposed view *Nostoc* sp. R76DM phycocyanin (N-PC) and *Microchaete diplosiphon* phycocyanin (MD-PC) (PDB ID: 1CPC) for amino acids substitutions in N-PC. The N-PC and MD-PC are presented in green and red color, respectively. (**D**) Substitution Phe → Tyr at α18 is compensated by Glu → Asp at β87 position. (**E**) Substitution Phe → Trp at β18 is compensated by Met → Ala at β79 position. (**F**) Substitution Ala → Val at β56 is compensated by two substitutions, Leu → Ile at β83 and Met → Leu at β86 position.

The overall 3-dimensional structure of N-PC is similar to existing PC structures. In N-PC structure, the α- and β-subunits interact through their N-terminal helices (buried area 6810 Å^2^) with 69.8 kcal mol^−1^ solvation free energy gain to forms αβ heterodimer (hereafter αβ monomer) (Fig. 3A). Three such αβ monomers join to form α_3_β_3_ trimer (Fig. 3B) and two such trimers pack in a face-to-face manner to form (α_3_β_3_)_2_ hexamer resulting in a biologically active PC assembly (Fig. 3C). Altogether, the N-PC hexamer consists of 12 protein chains (six α- and six β-subunits) and 18 PCBs adopting a doughnut like structure in a manner that all α-subunits are sandwiched between two layers of β-subunit with a gain of −486.7 kcal mol^−1^ solvation free energy. Each α-subunit contains one (αPCB1163) and β-subunit contains two (βPCB1173 and βPCB1174) covalently attached PCB molecules, adopting an anti-syn-anti conformation. Table 3 shows closest possible distances between αPCB1163, βPCB1173 and βPCB1174 within the αβ monomer, α_3_β_3_ trimer and (α_3_β_3_)_2_ hexamer. These values suggest that the closest distance between any two PCBs is too large (>35 Å) in αβ monomer for energy transfer. The oligomerization of αβ monomer in to trimer and hexamer brings PCB of neighboring monomer closer (~20–25 Å) and facilitates the transfer of energy.

**Table 3:** The possible closest distances between chromophores in *Nostoc* sp. R76DM phycocyanin αβ monomer, α_3_β_3_ trimer and (α_3_β_3_)_2_ hexamer assembly.

Assembly	Distance (Å) between					
	α1163 and β1173	β1173 and β1174	B1174 and α1163	Inter α1163	Inter β1173	Inter β1174
αβ monomer	50.9	38.7	50.0	>51.0	>51.0	>51.0
α_3_β_3_ trimer	20.2	38.7	38.0	>51.0	33.3	>51.0
(α_3_β_3_)_2_ hexamer	20.2	38.7	38.0	26.8	33.1	27.5

#### Compensatory nature of substitutions found in N-PC

Mapping of substitutions on N-PC 3D structure revealed that substitutions are of ‘compensatory’ nature. The substitution αPhe18 → αTyr18 is compensated by βGlu87 → βAsp87 substitution. The shortened side chain of βAsp87 helps hydroxyl group of αTyr18 to accommodate and stabilize via H-bond (Fig. 3D). Similarly, βPhe60 → βTrp60 substitution is compensated by βMet79 → βAla79 as the bulky side chain of βTrp60 occupies the empty space created by substitution of βMet79 with βAla79 (Fig. 3E). Furthermore, in substitution of βAla56 → βVal56, an addition of two methyl group is compensated by substitutions, βLeu83 → βIle83 and βMet86 → βLeu86 as shown in Fig. 3F.

#### Chromophore geometry and its interaction with protein micro-environment

**Figure 4:**
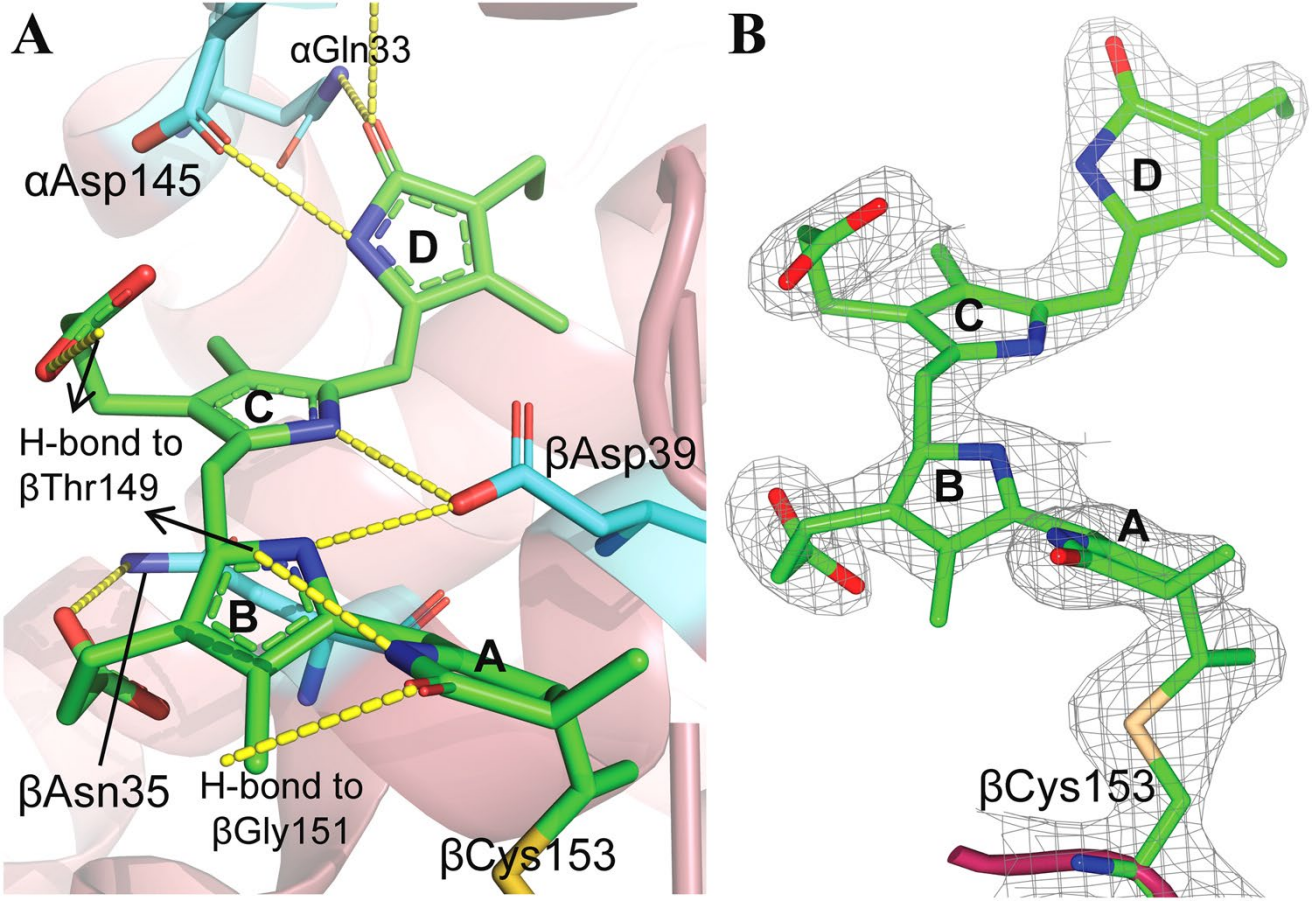
(**A**) Binding site of chromophore βPCB1174 in *Nostoc* sp. R76DM phycocyanin protein matrix. H-bonds are represented as yellow-dashed line. (**B**) Electron density map fit of a chromophore βPCB1175 (attached covalently to βCys153) in (2Fo-Fc) map drawn at 1.5σ contour level. Figure was prepared using PyMol and CCP4MG software.

The N-PC contains three PCB chromophores; one is attached covalently with Cys84 of α-subunit (αPCB1163), and two chromophores attached to Cys82 and Cys153 of β-subunit (βPCB1173 and βPCB1174, respectively). As per convention used in Peng *et al*.^8^, PCB attaches with conserved Cys residues through the pyrrole ring, called A-ring, which lacks π-conjugation unlike PCB’s subsequent (B, C and D) rings (Suppl. Material III). The geometry of αPCB1163 and βPCB1173 tetrapyrrole rings and their interactions with apoprotein in N-PC structure are nearly similar to other PC structures (Suppl. Material IV and V). Whereas, the βPCB1174 acquired unique conformation in N-PC; its B- and C- pyrrole ring deviate from co-planarity by an angle of 31.71 ± 5.09°, which is significantly higher as compared to that in reported PC structures (Suppl. Material VI). The binding pocket of βPCB1174 in N-PC is shown in Fig. 4. The βPCB1174 is located towards periphery of PC-hexamer and more solvent-exposed as compared to other two PCBs. The orientation of βPCB1174 is mainly controlled by the covalent bond with Cys153 and large network of hydrophobic interactions with surrounding residues (Suppl. Material V). Ring A of βPCB1174 is held via two H-bonds to main chain (β-chain) along with a covalent bond with βCys153 (Fig. 4, Suppl. Material IV). Ring B is less solvent exposed as compared to C-ring and masked by a protein loop made up of residues β146-β152, in which βThr149 is closest residue and likely to influence the orientation of B-ring. The orientation of rings B and C is favored by two sets of H-bonds; H-bond of propionic acid –COOH groups with conserved βAsn35 and βThr149 residues, respectively and H-bond of pyrrole ring protonated N-atoms with conserved βAsp39 (Fig. 4, Suppl. Material IV). Ring-D is surrounded by α-chain residues, αPhe28, αGln33 and αAsp145. The αPhe28 phenyl ring pushes D-ring away above the C-ring plane and this orientation of D-ring is stabilized by H-bonds with αGln33 and αAsp145 side chains (Fig. 4, Suppl. Material IV). As discussed in Gupta *et al*.^17^, occurrence of these three residues is co-linked with each other and with a specific orientation of D-ring. Residues directly interacting with βPCB1174 in N-PC structure are nearly conserved among all PC structures (Suppl. Material IV).

### *In vitro* and *in vivo* antioxidant activity of N-PC

Since PC has been widely reported to have antioxidant activity, effect of substitutions on N-PC’s antioxidant activity was checked through *in vivo* and *in vitro* antioxidant assays. Results of N-PC’s antioxidant activity assays are summarized and compared with other PC in Table 4. *In vitro* antioxidant assays (DPPH, FRAP and reducing power) showed that N-PC possess substantial antioxidant potential and these are comparable to the other PCs. Similarly, *in vivo* assay suggested that N-PC can efficiently avert stress-induced ROS generation and physiological abnormalities in *C*. *elegans* like other PC.

**Table 4:** *In vitro* and *in vivo* antioxidant activity of *Nostoc* sp. R76DM phycocyanin and its comparative account with other phycocyanin.

		*Nostoc* sp. R76DM	*Synechococcus* sp. R42DM^33^	*Leptolyngbya* sp. N62DM^31^	*Phormidium* sp. A09DM^9^
*In vitro* (Biochemical assay)	DPPH assay (% DPPH scavenging)	63.15 ± 0.85	71.10 ± 0.06	74.85 ± 2.14%	72.26 ± 1.21%
	FRAP assay (Ascorbic acid equivalent antioxidant Capacity)	0.22 ± 0.01	0.29 ± 0.05	0.49 ± 0.04	0.46 ± 0.06
	Reducing power assay (Ascorbic acid equivalent antioxidant Capacity)	0.40 ± 0.01	0.44 ± 0.03	0.53 ± 0.05	0.48 ± 0.04
*In vivo* (*C. elegans*)	DCFH-DA staining (% reduction in fluorescence intensity)	88.70	78.88	83.11	Not available
	Protective effect against oxidative stress (Fraction survival in %)	84.04 ± 6.27	80.00 ± 7.07	67.2 ± 6.40	64.9 ± 5.10
	Protective effect against thermal stress (Increase in fraction survival in %)	~36	~10	~25	Not available

## Discussion

Recently, Rastogi *et al*.^14^ has identified a monomeric allophycocyanin in *Nostoc* sp. R76DM having significantly blue-shifted spectral property. Since allophycocyanin receives energy from PC, we intended to study PC of this organism. Two distinct features of N-PC, twelve substitutions across invariant sequence (Table 1) and a blue-shifted spectral properties (Fig. 1B), were investigated using physico-chemical, phylogenetic and structural analysis.

Out of 334 residues of PC α- and β-subunit, almost 31% residues are strictly conserved and ~69% residues/positions keep varying among cyanobacterial species. Available PC structures suggest that most of the conserved (~31%) residues are involved in crucial interactions like chromophore-protein, monomer-monomer or trimer-trimer interactions. Substitutions, observed in N-PC are recognized as a distinctive sequence feature of other *Nostoc* PC. This suggests that these substitutions might have occurred under an evolutionary pressure associated with *Nostoc* species. Positions of these substitutions in N-PC trimer and hexamer have been mapped onto the structure to understand significance of these substitutions. In N-PC (αβ)_3_ trimer, substituting residues are congregated at the interface of two αβ monomer. Out of 9 substitutions in β-subunit, six (βVal56, βTrp60, βAla79, βIle83, βLeu86 and βLeu134) occur along an interactive length of three alpha-helices in a way that maintains helix-helix interaction, also termed as a ‘knobes into holes’ interactions required to maintain topology of globular protein^18^. The requirement of helix-helix packing to preserve β-subunit topology thus might have acted as functional pressure for these substitutions. The pair of substitutions, αTyr18 and βAsp187 make an additional H-bond between α- and β-subunit of adjacent αβ monomer within trimer. This pair might provide additional stability to N-PC trimer assembly to maintain light harvesting under extreme environmental conditions. The substitution βAla109 is present on F’ helix placed towards the central cavity of hexameric assembly. As per the recent report of Zhang *et al*.^19^, rod linker (L_R1_) protein mainly interacts with F and F’ helices of β-subunit. Since substituted residue βAla109 is present on likely binding site for linker protein, a need to maintain interaction with linker protein might be a pressure for occurrence of this substitution. Structural implications of remaining three substitutions, αVal88, αAla98 and βSer138 could not be identified, however.

The absorption spectrum of all three PCB of N-PC should be identical in free form due to their identical chemical nature. However, in protein-bound form, the absorption characteristic of PCB may differ due to their differently constrained geometry and that is why PC absorption spectrum bears a composite rather than pure nature. The absorption spectra of protein-embedded PCB are a relative function of conjugation between its B-C-D rings^8^. The extent of conjugation depends on the co-planarity between these (B-C-D) rings and represented by an angle of deviation between them^8^. From available PC structures, it is inferred that the B- and C-rings of PCB are nearly co-planner (angle <15° in most cases, and <24° in all cases) (Suppl. Material VI), whereas ring D deviates significantly from the B-C plane. The deviation of D-ring from B-C plane decreases the effective π-conjugation length of PCB and cause a blue-shift in absorbance of PC^8,15^. Deconvolution of N-PC absorption spectra suggested that the blue-shift in its absorption is a collective effect of blue shifts in its deconvoluated components 1 and 2 (Fig. 1C). Each component of N-PC absorption shown in Fig. 1C should be a result of contributions from all three PCBs; however, the magnitude of contribution may differ. As per the report of Demidov and Mimuro^15^, chromophores βPCB1174 and αPCB1163 contributes majorly to 598 and 618 nm components, respectively; whereas, βPCB1173 contributes to 625 nm component. Analysis of PCB geometry in N-PC suggested that the conjugation of βPCB1174 is reduced uniquely due to loss of planarity between its B- and C-ring planes. Since the decreased conjugation length is associated with spectral blue-shift, the component 1 of N-PC spectra should be blue-shifted due to the unique conformation of the βPCB1174. Comparable smaller blue-shift in component 2 may be due to fusion effect of blue-shifted βPCB1174 and αPCB1163 absorption components.

Presently described N-PC structure would provide a template for confident modeling of other PC of *Nostoc* clade. Like other PC, N-PC also possesses significant antioxidant potential and is promising nutraceutical and pharmaceutical molecule. The bio-physical properties and structural information of N-PC described in the present report would be useful in developing biomedical application of N-PC.

## Materials and Methods

### Cyanobacterial culture and growth conditions

The fresh water cyanobacterium *Nostoc* sp. R76DM (accession number KJ994254) was cultivated in BG11^20^ liquid medium at 27 ± 2 °C with 12:12 h light:dark cycles under 12 W m^−2^ cool white fluorescence illumination as described previously^14^.

### Extraction and purification of N-PC

#### Cell lysis and PBPs extraction

The cell mass was harvested and washed with extraction buffer (20 mM Tris-HCl buffer, pH 8.0). For extraction of PBPs from cell, first, an ultra-sonication (Sonics Vibra Cell, Sonics and Material Inc, USA) method was employed, which was followed by two freeze-thaw cycles at −20 °C and 4 °C, respectively. The cell debris was removed from crude extract by centrifugation at 19,320 × *g* for 10 min at 4 °C (KUBOTA 6500, Japan).

#### Ammonium sulphate precipitation and chromatography

The crude extract was subjected to 20–70% ammonium sulphate precipitation to get PC retained in precipitate. The pellet of precipitate, obtained after centrifugation (19,320 × *g*, 10 min, 4 °C, KUBOTA 6500, Japan), was dissolved in 20 mM Tris-HCl buffer, pH 8.0 and desalted by dialysis against same buffer. Dialyzed N-PC was further purified by weak anion exchange (DEAE-cellulose) and gel permeation (Superdex 200) chromatography.

### Physico-chemical characterization of N-PC

#### SDS-PAGE analysis

SDS-PAGE was performed to check the purity of N-PC preparation as described previously^9^.

#### UV-visible and fluorescence emission spectroscopy

The absorption spectrum of purified PC was recorded by UV-visible spectrophotometer (V-630, JASCO) over the range of 250 to 750 nm. The emission spectrum was recorded by fluorescence spectroscopy (FP-8500, JASCO) using excitation wavelength of 580 nm. The spectroscopic measurement was conducted at room temperature. UV-visible spectra were analyzed and doconvoluated through OriginPro8 software (OriginLab, Northampton, MA).

### Sequence determination and phylogenetic analysis

The gene sequences of *cpcA* and *cpcB*, encoding α- and β-subunits of N-PC, were deduced from whole genome sequence (unpublished) and submitted to NCBI GenBank with an accession no. MK561022 and MK561023 respectively. The retrieval, multiple sequence alignment of phycocyanin protein sequences and its representation were performed using NCBI-BLAST, Clustal Omega and ESPript^21^, respectively. The 16s rRNA cyanobacterial sequences were also retrieved from NCBI. The phylogenetic and molecular evolution analyses of protein and 16S rRNA gene sequences were conducted using MEGA X software^22^. The evolutionary history was inferred using Maximum-likelihood and JTT matrix-based method^23^. The bootstrap consensus tree inferred from 50 replicates was taken to represent evolutionary history. Relative divergence rates of α- and β-subunits were estimated by RealTime-ML function of MEGA X software.

### Crystallographic analysis

#### Crystallization and data collection

Crystallization trials for N-PC (10 mg mL^−1^) were performed by sitting drop vapor diffusion method using pre-formulated commercial screens JCSG+ and PACT, obtained from Qiagen. Initial responses were further optimized and diffraction quality crystals of N-PC were raised with solution 0.2 M sodium malonate and 20% (w/v) PEG 3350 at 25 °C. Bigger crystals were picked up in LithoLoops (Molecular Dimension), flash-cooled and stored in liquid nitrogen with glycerol as a cryo-protectant. Stored crystals were shot at PX BL-21 beam line, INDUS-2 synchrotron radiation facility, India, and diffraction intensity data were collected on image plate reader detector (MarXperts)^24^.

#### Data processing and analysis of 3-D structure

Collected data was integrated in XDS program^25^; and scaled, merged and truncated using Aimless and Ctruncate program of CCP4 suite^26^. Initial phases for N-PC crystals were obtained through molecular replacement method by *PHASER*^27^ using *Gracilaria chilensis* PC structure (PDB ID: 2bv8) as a search model. The initial model was further refined through combination of automatic refinement by *Phenix*^28^ and manual refinement by COOT^29^, until the reasonable R-factors and stereo-chemistry of model was achieved. The stereo-chemistry of model was monitored during refinement by MOLPROBITY software^30^. Refined coordinates and structure factors have been submitted to protein data bank with ID 6JPR. The 3D representation of structural figures were prepared using PYMOL package (LLC Schrodinger).

### *In vitro* and *in vivo* antioxidant activity of N-PC

Antioxidant assays, 2,2-diphenyl-1-picrylhydrazyl (DPPH)-radical scavenging activity, ferric ion reducing ability of plasma (FRAP) and reducing power (RP) assay were performed as described earlier^31^. The *in*-*vivo* antioxidant activity was assessed using *C*. *elegans* as a model organism. The age synchronized worms were used to analyze the effect of oxidative stress (100 mM H_2_O_2_ treatment for 2 h) and thermal stress (incubation at 35 °C temperature) on N-PC treated worms (100 μg/ml N-PC containing plate) and untreated worms^32^. The intra-cellular ROS level in 10 mM paraquat exposed worms was analyzed by staining the treated and control worms with 2,7-dichlorodihydrofluoroscein diacetate (DCFH-DA) stain and visualized under fluorescence microscope (BX-41, Olympus, Japan) as described earlier^32^.

## Supplementary information

supplementary material

## Data Availability

The co-ordinates associated with the present study are available at the Protein Data Bank with PDB ID 6JPR. The *cpcA* and *cpcB* gene sequences of *Nostoc* sp. R76DM are available at NCBI database with accession nos MK561022 and MK561023 respectively.
