# Supplementary material for: Phylogenetic and crystallographic analysis of Nostoc phycocyanin having blue-shifted spectral properties
Source: Sci Rep. 2019 Jul 8;9:9863. doi: 10.1038/s41598-019-46288-4 (PMC6614406; doi:10.1038/s41598-019-46288-4)
Supplement: Supplementary file 1 — supplementary material [file 41598_2019_46288_MOESM1_ESM.docx]

**Supplementary Materials**

of

Phylogenetic and crystallographic analysis of *Nostoc* phycocyanin having blue-shifted spectral properties

Ravi R. Sonani^1^, Rajesh Prasad Rastogi^2^, Stuti Nareshkumar Patel^3^, Mukesh Ghanshyam Chaubey^4^, Niraj Kumar Singh^4^, Gagan D. Gupta^1^, Vinay Kumar^1,*^, Datta Madamwar^3,*^

^1^Radiation Biology & Health Sciences Division, Bhabha Atomic Research Centre, Trombay, Mumbai - 400 085, India

^2^Ministry of Environment, Forest & Climate Change, Indira Paryavaran Bhawan, New Delhi-110003, India.

^3^Post-Graduate Department of Biosciences, Satellite Campus, Sardar Patel University, Bakrol, Anand 388 315, Gujarat, India

^4^Shri A. N. Patel P. G. Institute of Science and Research, Sardar Patel University, Anand, Gujarat, India

*Corresponding authors

1. Mail: [vinay@barc.gov.in,](mailto:vinay@barc.gov.in,) [datta_madamwar@yahoo.com](mailto:datta_madamwar@yahoo.com)

**Supplementary Material I**

**Table**: Summary of available phycocyanin 3D structures in the Protein Data Bank and protein spectral characteristics

| **PDB ID** | **Organism** | **Resolution**  **(Å)** | **Absorption maximum**  **(nm)** | **Emission maximum**  **(nm)** | **Reference** |
| --- | --- | --- | --- | --- | --- |
| 2UUM | *Arthrospira platensis* | 3.0 | ~618-620 | ~650 | (Patel et al., 2004)^1^ |
| 4L1E | *Leptolyngbya* sp. N62DM | 2.61 | 620 | Not available | (Singh et al., 2014)^2^ |
| 1GH0 | *Arthrospira platensis* | 2.20 | ~618-620 | ~650 | (Wang et al., 2001)^3^ |
| 1HA7 | *Arthrospira platensis* | 2.20 | ~618-620 | ~650 | (Padyana et al., 2001)^4^ |
| 2UUL | *Spirulina* sp. | 3.1 | ~618-620 | Not available | (Patel et al., 2004)^1^ |
| 2UUN | *Leptolyngbya* sp. | 3 | ~618-620 | Not available | (Patel et al., 2004)^1^ |
| 5OOK | *[Acaryochloris marina](https://www.rcsb.org/pdb/search/smartSubquery.do?smartSearchSubtype=TreeEntityQuery&t=1&n=329726)* [(strain MBIC 11017)](https://www.rcsb.org/pdb/search/smartSubquery.do?smartSearchSubtype=TreeEntityQuery&t=1&n=329726) | 2.1 | 616-618 | 645 | (Bar-Zvi et al. 2018)^5^ |
| 5TOU | *[Pseudanabaena](https://www.rcsb.org/pdb/search/smartSubquery.do?smartSearchSubtype=TreeEntityQuery&t=1&n=1357935)* [sp. lw0831](https://www.rcsb.org/pdb/search/smartSubquery.do?smartSearchSubtype=TreeEntityQuery&t=1&n=1357935) | 2.04 | 620 | 640 | (Su et al., 2017)^6^ |
| 1CPC | *[Microchaete diplosiphon](https://www.rcsb.org/pdb/search/smartSubquery.do?smartSearchSubtype=TreeEntityQuery&t=1&n=1197)* | 1.66 | 614 | 644 | (Duerring et al., 1991)^7^ |
| 4YJJ | *Phormidium rubidium* | 2.7 | 616 | 645 | (Gupta et al., 2016)^8^ |
| 4F0T | *[Synechocystis](https://www.rcsb.org/pdb/search/smartSubquery.do?smartSearchSubtype=TreeEntityQuery&t=1&n=1111708)* [sp. (strain PCC 6803/Kazusa)](https://www.rcsb.org/pdb/search/smartSubquery.do?smartSearchSubtype=TreeEntityQuery&t=1&n=1111708) | 2.25 | 620 | 650 | (Marx and Adir, 2013)^9^ |
| 4H0M | *Synechococcus elongatus* (strain PCC 7942) | 2.20 | 618 | 645 | (Marx and Adir, 2013)^9^ |
| 1F99 | *[Polysiphonia urceolata](https://www.rcsb.org/pdb/search/smartSubquery.do?smartSearchSubtype=TreeEntityQuery&t=1&n=65404)* | 2.4 | 549, 617 | 636 | (Jiang et al., 2001) (Wang et al., 2014)^10,11^ |
| 2BV8 | *[Gracilaria tenuistipitata var. liui](https://www.rcsb.org/pdb/search/smartSubquery.do?smartSearchSubtype=TreeEntityQuery&t=1&n=285951)* | 2.01 | 621 | Not available | (Contreras-Martel et al., 2007)^12^ |
| 1PHN | *[Galdieria sulphuraria](https://www.rcsb.org/pdb/search/smartSubquery.do?smartSearchSubtype=TreeEntityQuery&t=1&n=130081)* | 1.65 | 618 | Not available | (Stec et al., 1999)^13^ |
| 3BRP | *[Galdieria sulphuraria](https://www.rcsb.org/pdb/search/smartSubquery.do?smartSearchSubtype=TreeEntityQuery&t=1&n=130081)* | 1.85 | 618 | Not available | (Moon et al., 2014)^14^ |
| 3l0F | *Thermosynechococcus elongatus* (strain BP-1) | 1.35 | 618 | Not available | RCSB Protein data bank |
| 1KTP | *[Thermosynechococcus elongatus](https://www.rcsb.org/pdb/search/smartSubquery.do?smartSearchSubtype=TreeEntityQuery&t=1&n=197221)* [(strain BP-1)](https://www.rcsb.org/pdb/search/smartSubquery.do?smartSearchSubtype=TreeEntityQuery&t=1&n=197221) (expressed in *E. coli*) | 1.6 | 618 | Not available | (Adir et al., 2002)^15^ |
| 3O18 | *Thermosynechococcus vulcanus* | 1.35 | 618 | Not available | (David et al., 2011)^16^ |
| 2VJR | *[Gloeobacter violaceus](https://www.rcsb.org/pdb/search/smartSubquery.do?smartSearchSubtype=TreeEntityQuery&t=1&n=251221)* [(strain ATCC 29082 / PCC 7421)](https://www.rcsb.org/pdb/search/smartSubquery.do?smartSearchSubtype=TreeEntityQuery&t=1&n=251221) | 2.6 | 620 | 648 | (Mimuro et al., 2002)^17^ |
| **6JPR** | ***Nostoc* sp. R76DM** | **2.35** | **611** | **635** | **Present study** |

**Supplementary Material II**

**Figure**: Multiple sequence alignment (MAS) of phycocyanin β-subunit amino acid sequences. Conserved residues are highlighted in red. Conserved motifs are enclosed in vertical blue boxes. The unique substitutions for *Nostoc* sp. R76DM phycocyanin are indicated by ‘#’. The figure was prepared using EsPript 3.0 online tool^18^.

**Supplementary Material III**

**Figure**: Chemical structure of phycocyanobilin chromophore attached to the conserved Cys residue through its A-ring in phycocyanin

**Supplementary Material IV**

Description of chromophores geometry and their interactions with surrounding apoprotein residues in *Nostoc* sp. R76DM phycocyanin

**The αPCB1163 chromophore**

**Figure A**: Binding sites of chromophore αPCB1163 in *Nostoc* sp. R76DM phycocyanin protein matrix. H-bonds are represented as yellow-dashed line. Figure was constructed in PyMol

**Table A**: Details of chromophore αPCB1163 interactions with surrounding apoprotein in *Nostoc* sp. R76DM phycocyanin structure

| **Chromophore** | **Type of Interaction** | **Involved PCB Atom** | **Bonded with (Atom, Residue, Chain)** |
| --- | --- | --- | --- |
| αPCB1163 | Covalent | Ring A, CAA | SG, Cys84, A |
|  | H-bond | Ring A, NA | N, Ala75, A |
|  |  | Ring A, OA | O, Asn73, A |
|  |  | Ring B, NB | OD2, Asp87, A |
|  |  | Ring B, O2B | NE, Arg57, F |
|  |  | Ring C, NC | OD1, Asp87, A |
|  |  | Ring C, O1C | NZ, Lys83, A |
|  |  | Ring C, O2C | NH1, Arg86, A |
|  |  | Ring C, O2C | NH2, Arg86, A |
|  |  | Ring D, OD | N, Thr75, F |

The chromophore αPCB1163 is positioned at the interface of two αβ-heterodimers (Fig. A). Position of αPCB1163 ring A is well fixed by covalent bond with αCys84, and two H-bond with α-main chain CO- and NH-group. Co-planarity of ring B and C is fixed by sets of H-bonds and π-stacking interactions. Two types of H-bonds holding B- and C-rings together in same plane by stretching them in opposite directions along X-axis are identified. Type I, H-bonds of B- and C-rings N-atoms with carboxylic group of highly conserved Asp87A (αAsp87). Type II, H-bonds between -COOH group of B- and C-ring propionic acid side chains with Lys83A (αLys83), Arg86A (αArg86) and Arg57F (βArg57) (Fig. A, Table A). The π-stacking interactions along Y-axis hold B- and C-rings in same plane. Deviation of ring-D is controlled through the H-bond with neighbouring β-main chain NH-group. The D-ring plane deviates from B-C ring plane by an angle of 34.03±2.02^°^.

**Supplementary Material IV (Contd.)**

**The βPCB1173 chromophore**

**Figure B**: Binding site of chromophore βPCB1173 in *Nostoc* sp. R76DM phycocyanin protein matrix. H-bonds are represented as yellow-dashed line. Figure was constructed in PyMol

**Table B**: Details of chromophore βPCB1173 interactions with surrounding apoprotein in *Nostoc* sp. R76DM phycocyanin structure.

| **Chromophore** | **Type of Interaction** | **Involved PCB Atom** | **Bonded with (Atom, Residue, Chain)** |
| --- | --- | --- | --- |
| βPCB1173 | Covalent | Ring A, CAA | SG, Cys82, B |
|  | H-bond | Ring A, NA | OD1, MeN72, B |
|  |  | Ring B, NB | OD2, Asp85, B |
|  |  | Ring B, O2B | NH1, Arg77, B |
|  |  | Ring B, O2B | NH2, Arg77, B |
|  |  | Ring C, NC | OD2, Asp85, B |
|  |  | Ring C, O1C | NH1, Arg84, B |
|  |  | Ring C, O1C | NH2, Arg84, B |

The βPCB1173 is positioned towards the hollow cavity in PC-hexamer. Binding pocket of βPCB1173 is shown in Fig. B. Similar to αPCB1163, ring A of βPCB1173 is also rigidly held via H-bond to methylated asparagine-72 residue of β-subunit (βMeN72) and main chain, respectively (Fig. B, Table B). Like αPCB1163, rings B and C coplanarity in βPCB1173 is also achieved by a set of balanced H-bond along X-axis and π-stacking interactions along Y-axis (Fig. B). Unlike αPCB1163, ring D of βPCB1173 does not form any H-bond to protein; however, its deviation (of 42.32±3.87^o^) from B-C ring plane is achieved by network of hydrophobic contacts (See Suppl. Item V). Since, the linker proteins occupy central hollow cavity, the conformation βPCB1173 ring-D might be further modified upon binding of linker proteins.

**Supplementary Material IV (Contd.)**

**The βPCB1174 chromophore**

**Table C**: Details of chromophore βPCB1174 interactions with surrounding apoprotein in *Nostoc* sp. R76DM phycocyanin structure. The conserved residues, involved interaction is highlighted in bold. ‘x’ stands for non-conserved position

| **Type of Interaction** | **Involved PCB Atom** | **Bonded with (Atom, Residue, Chain)** | **Conserved motif** |
| --- | --- | --- | --- |
| Covalent | Ring A, CAA | SG, Cys153, B | 1. GD**C** -153 |
| H-bond | Ring A, NA | N, Gly151, B | 151- GD**C** -153 |
|  | Ring A, OA | O, Thr149, B | 1. G(I/T)(**T/P**)x -150 |
|  | Ring B, NB | OD2, Asp39, B | 39- **D**xVN -42 |
|  | Ring B, O2B | ND2, Asn35, B | 35- **N**KR -37 |
|  | Ring C, NC | OD2, Asp39, B | 39- **D**xVN -42 |
|  | Ring C, O1C | OG1, Thr149, B | 1. G(I/**T**)(T/P)x -150 |
|  | Ring D, ND | OD1, Asp145, G | 1. L(S/T)G(**D**/Q) -145 |
|  | Rind D, OD | NE2, Gln33, G | 31- (F/Y)x(R/**Q**)A -34 |

**Supplementary Material V**

**Figure**: The 2-D LigPlot+ diagrams of αPCB1163 (**A**), βPCB1173 (**B**) and βPCB1174 (**C**) binding pockets in *Nostoc* sp. R76DM phycocyanin structure showing network of the chromophore-protein hydrophobic contacts. Chromophore is represented by ball-stick model with blue bond colour. Residues forming covalent and H-bonds with the chromophore atoms are shown as ball-sticks model with black bond colour. Residues making only hydrophobic contact(s) are represented by brick-red colour (radiating) partial circle along with residue labels. Covalent bond C_PEB_-S_Cys_ is indicated with purple color. Carbon, nitrogen, oxygen and sulphur atoms are represented by grey, green, red and yellow balls, respectively. The figure was constructed using LigPlot+ suite^19^.


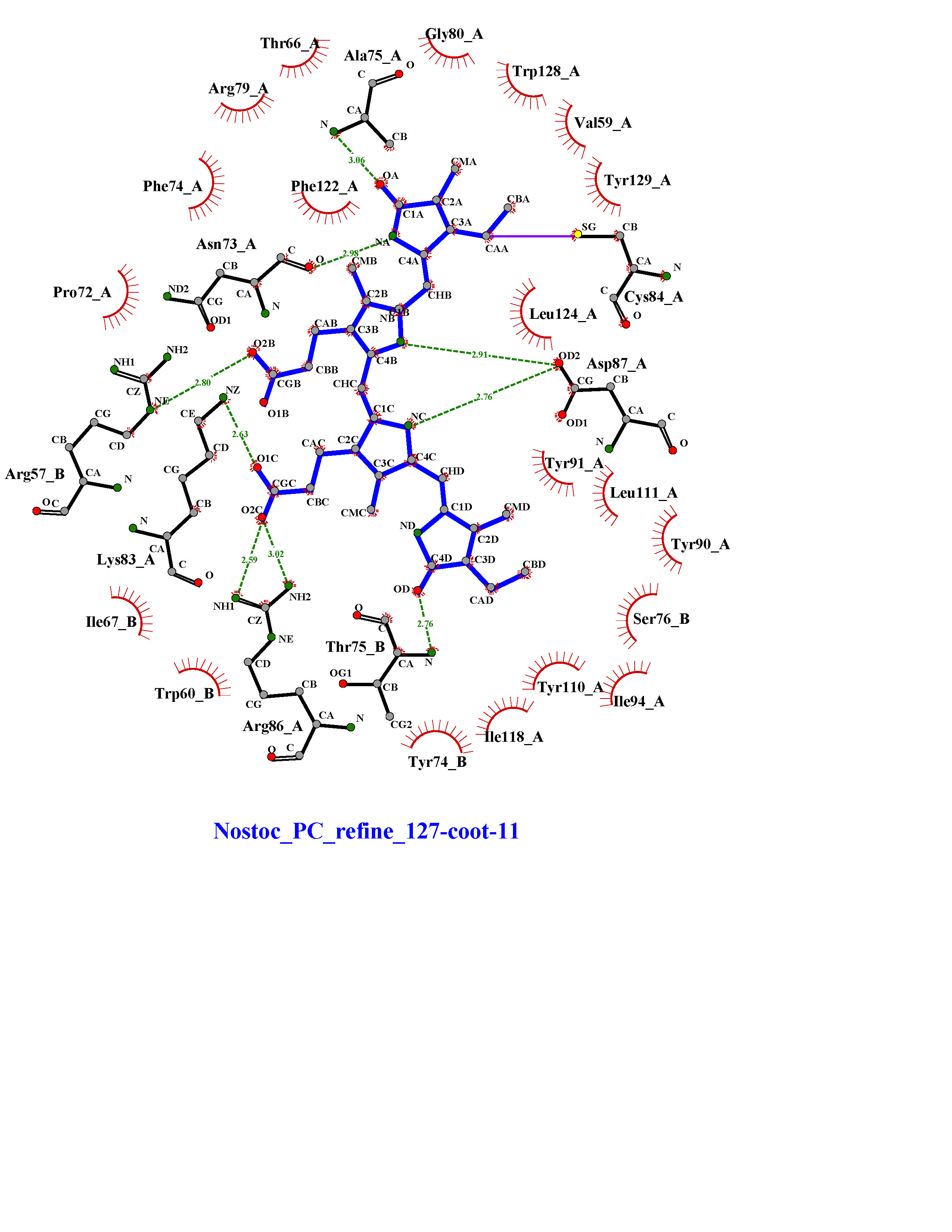


**A**

**Supplementary Material V (Contd.)**


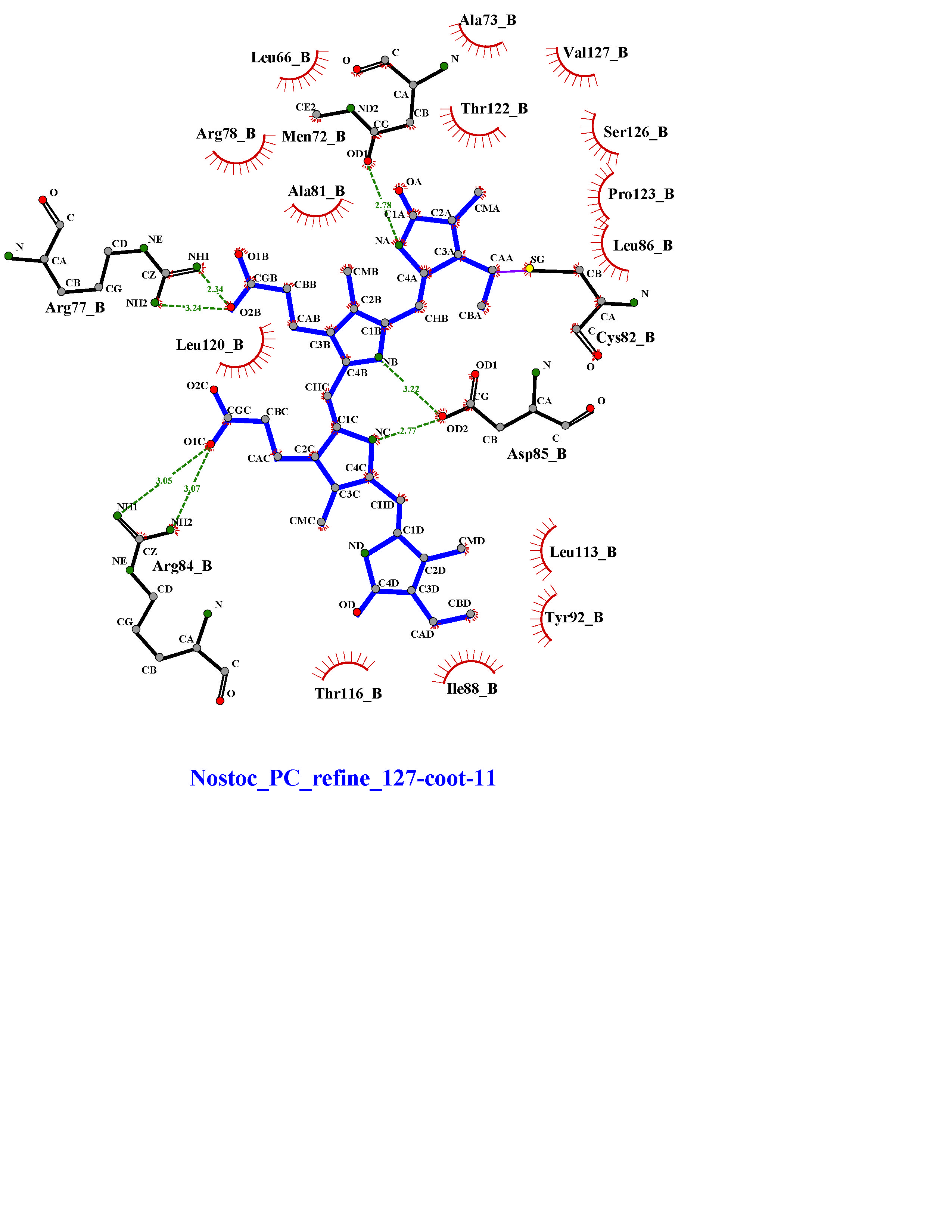


**B**

**Supplementary Material V (Contd.)**

**Supplementary Material VI**


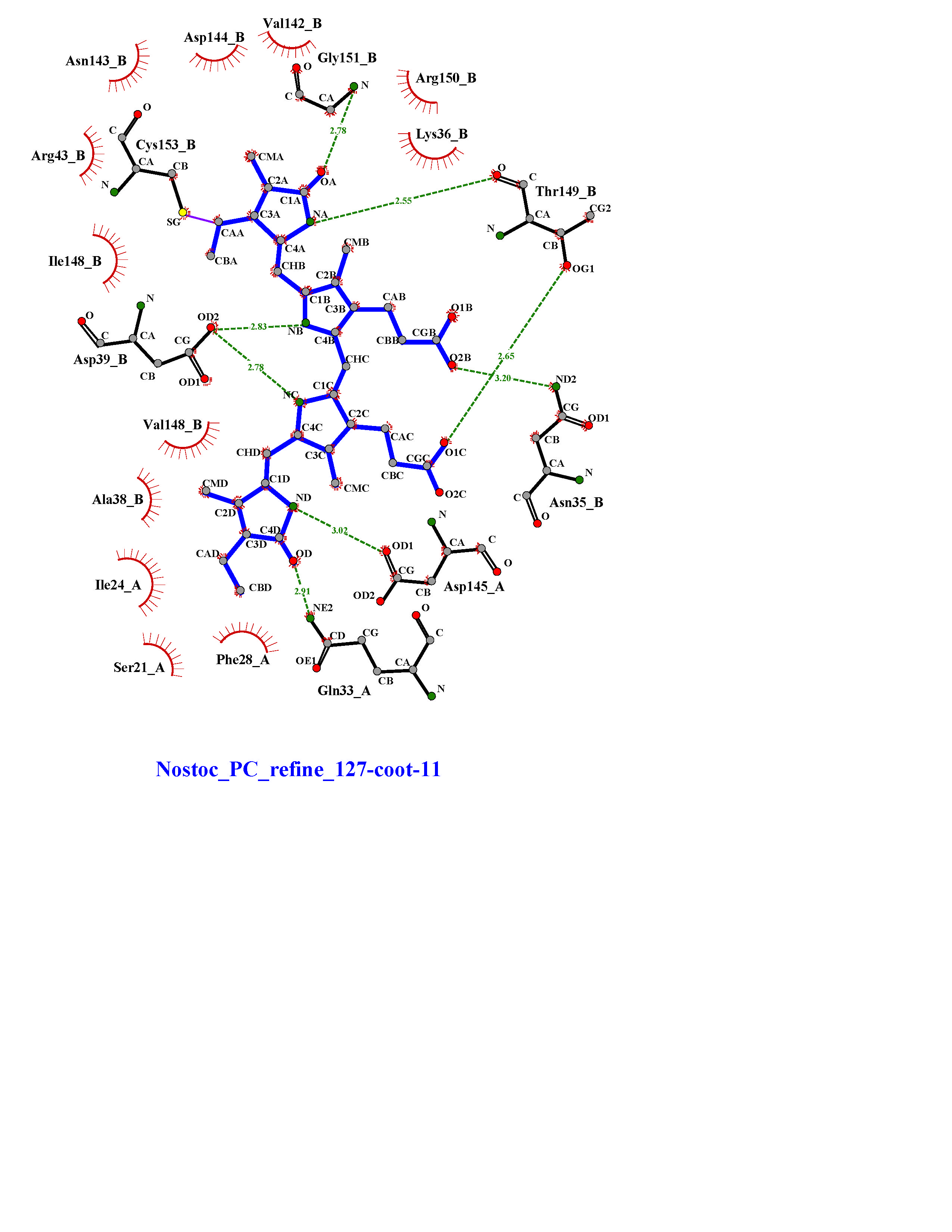


**C**

**C**

**Table**: The angle of deviation between B- and C-rings planes of βPCB1173 chromophore of phycocyanin structures available in the Protein Data Bank

| **PDB ID** | **Angle of deviation between B- and C- rings (degrees))** |
| --- | --- |
| 2uum | 0.8 |
| 4l1e | 23.6 |
| 1gh0 | 8.0 |
| 1ha7 | 7.2 |
| 2uul | 1.1 |
| 2uun | 23.7 |
| 5ook | 10.6 |
| 5tou | 4.6 |
| 1cpc | 9.2 |
| 4yjj | 6.5 |
| 4f0t | 10.3 |
| 4h0m | 3.7 |
| 1f99 | 17.2 |
| 2bv8 | 5.8 |
| 1phn | 12.5 |
| 3brp | 14.4 |
| 3l0f | 16.5 |
| 1jbo | 5.8 |
| 1ktp | 20.0 |
| 3o18 | 7.9 |
| 2vjr | 3.2 |
| 6jpr (Present Study) | 31.7 |

**References**

1. Patel, A., Mishra, S., Pawar, R. & Ghosh, P. K. Purification and characterization of C-Phycocyanin from cyanobacterial species of marine and freshwater habitat. *Protein Expr. Purif.* **40**, 248–255 (2005).

2. Singh, N. K. *et al.* Crystal structure and interaction of phycocyanin with β-secretase: A putative therapy for Alzheimer’s disease. *CNS Neurol. Disord. Drug Targets* **13**, 691–698 (2014).

3. Wang, X.-Q. *et al.* Structure of C-phycocyanin from Spirulina platensis at 2.2 Å resolution: a novel monoclinic crystal form for phycobiliproteins in phycobilisomes. *Acta Crystallogr. D Biol. Crystallogr.* **57**, 784–792 (2001).

4. Padyana, A. K., Bhat, V. B., Madyastha, K. M., Rajashankar, K. R. & Ramakumar, S. Crystal Structure of a Light-Harvesting Protein C-Phycocyanin from Spirulina platensis. *Biochem. Biophys. Res. Commun.* **282**, 893–898 (2001).

5. Bar-Zvi, S. *et al.* Structural heterogeneity leads to functional homogeneity in A. marina phycocyanin. *Biochim. Biophys. Acta BBA - Bioenerg.* **1859**, 544–553 (2018).

6. Su, H.-N. *et al.* Structural insights into the cold adaptation of the photosynthetic pigment-protein C-phycocyanin from an Arctic cyanobacterium. *Biochim. Biophys. Acta BBA - Bioenerg.* **1858**, 325–335 (2017).

7. Duerring, M., Schmidt, G. B. & Huber, R. Isolation, crystallization, crystal structure analysis and refinement of constitutive C-phycocyanin from the chromatically adapting cyanobacterium Fremyella diplosiphon at 1.66 Å resolution. *J. Mol. Biol.* **217**, 577–592 (1991).

8. Gupta, G. D. *et al.* Crystal structure analysis of phycocyanin from chromatically adapted Phormidium rubidum A09DM. *RSC Adv.* **6**, 77898–77907 (2016).

9. Marx, A. & Adir, N. Allophycocyanin and phycocyanin crystal structures reveal facets of phycobilisome assembly. *Biochim. Biophys. Acta BBA - Bioenerg.* **1827**, 311–318 (2013).

10. Jiang, T., Zhang, J., Chang, W. & Liang, D. Crystal Structure of R-Phycocyanin and Possible Energy Transfer Pathways in the Phycobilisome. *Biophys. J.* **81**, 1171–1179 (2001).

11. Wang, Y., Gong, X., Wang, S., Chen, L. & Sun, L. Separation of Native Allophycocyanin and R-Phycocyanin from Marine Red Macroalga Polysiphonia urceolata by the Polyacrylamide Gel Electrophoresis Performed in Novel Buffer Systems. *PLOS ONE* **9**, e106369 (2014).

12. Contreras-Martel, C. *et al.* The structure at 2 Å resolution of Phycocyanin from Gracilaria chilensis and the energy transfer network in a PC–PC complex. *Biophys. Chem.* **125**, 388–396 (2007).

13. Stec, B., Troxler, R. F. & Teeter, M. M. Crystal Structure of C-Phycocyanin from Cyanidium caldarium Provides a New Perspective on Phycobilisome Assembly. *Biophys. J.* **76**, 2912–2921 (1999).

14. Moon, M. *et al.* Isolation and characterization of thermostable phycocyanin from Galdieria sulphuraria. *Korean J. Chem. Eng.* **31**, 490–495 (2014).

15. Adir, N., Vainer, R. & Lerner, N. Refined structure of c-phycocyanin from the cyanobacterium Synechococcus vulcanus at 1.6 Å: insights into the role of solvent molecules in thermal stability and co-factor structure. *Biochim. Biophys. Acta BBA - Bioenerg.* **1556**, 168–174 (2002).

16. David, L., Marx, A. & Adir, N. High-Resolution Crystal Structures of Trimeric and Rod Phycocyanin. *J. Mol. Biol.* **405**, 201–213 (2011).

17. Mimuro, M. *et al.* Unique Fluorescence Properties of a Cyanobacterium Gloeobacter violaceus PCC 7421: Reasons for Absence of the Long-Wavelength PSI Chl a Fluorescence at –196°C. *Plant Cell Physiol.* **43**, 587–594 (2002).

18. Robert, X. & Gouet, P. Deciphering key features in protein structures with the new ENDscript server. *Nucleic Acids Res.* **42**, W320–W324 (2014).

19. Laskowski, R. A. & Swindells, M. B. LigPlot+: multiple ligand-protein interaction diagrams for drug discovery. *J. Chem. Inf. Model.* **51**, 2778–2786 (2011).
